# Machine learning for identifying randomised controlled trials when conducting systematic reviews: Development and evaluation of its impact on practice

**DOI:** 10.1017/rsm.2025.3

**Published:** 2025-03-21

**Authors:** Xuan Qin, Minghong Yao, Xiaochao Luo, Jiali Liu, Yu Ma, Yanmei Liu, Hao Li, Ke Deng, Kang Zou, Ling Li, Xin Sun

**Affiliations:** 1 Institute of Integrated Traditional Chinese and Chinese Evidence-based Medicine Center and Cochrane China Center and MAGIC China Center, West China Hospital, Sichuan University, Chengdu, China; 2 NMPA Key Laboratory for Real World Data Research and Evaluation in Hainan, Chengdu, China; 3 Sichuan Center of Technology Innovation for Real World Data, Chengdu, China; 4 Sichuan University West China College of Public Health/West China Fourth Hospital, Chengdu, China

**Keywords:** conducting systematic review, ensemble learning, impact on practice, title and abstract screening

## Abstract

Machine learning (ML) models have been developed to identify randomised controlled trials (RCTs) to accelerate systematic reviews (SRs). However, their use has been limited due to concerns about their performance and practical benefits. We developed a high-recall ensemble learning model using Cochrane RCT data to enhance the identification of RCTs for rapid title and abstract screening in SRs and evaluated the model externally with our annotated RCT datasets. Additionally, we assessed the practical impact in terms of labour time savings and recall improvement under two scenarios: ML-assisted double screening (where ML and one reviewer screened all citations in parallel) and ML-assisted stepwise screening (where ML flagged all potential RCTs, and at least two reviewers subsequently filtered the flagged citations). Our model achieved twice the precision compared to the existing SVM model while maintaining a recall of 0.99 in both internal and external tests. In a practical evaluation with ML-assisted double screening, our model led to significant labour time savings (average 45.4%) and improved recall (average 0.998 compared to 0.919 for a single reviewer). In ML-assisted stepwise screening, the model performed similarly to standard manual screening but with average labour time savings of 74.4%. In conclusion, compared with existing methods, the proposed model can reduce workload while maintaining comparable recall when identifying RCTs during the title and abstract screening stages, thereby accelerating SRs. We propose practical recommendations to effectively apply ML-assisted manual screening when conducting SRs, depending on reviewer availability (ML-assisted double screening) or time constraints (ML-assisted stepwise screening).

## Highlights

### What is already known?

- The standard method for retrieving randomised controlled trials (RCTs) from databases during systematic reviews (SRs) often results in only 7% of retrieved citations being real RCTs. Applying machine learning (ML) to identify RCTs is a promising solution.
- Existing ML models have seen limited use due to concerns about their performance and practical benefits, which have been questioned by SR practitioners.

### What is new?

- We developed a new ML model based on the Cochrane Crowd RCT dataset. This model demonstrated greater workload savings while maintaining a similar recall (0.99) to existing models in both internal and external evaluations.
- When used to assist in manual screening, our model enabled significant labour time savings and improved recall.

### Potential impact for readers of research synthesis methods?

- This study may be particularly beneficial for SR practitioners using ML methods to identify RCTs, as it offers a way to accelerate the conduct of SRs.
- Our developed model facilitates workload reduction while minimising the loss of cases, with ML-assisted manual screening offering a promising option for SR practitioners facing limited resources, such as a shortage of reviewers or time constraints.

## 1 Introduction

Systematic reviews (SRs) focused on randomised controlled trials (RCTs) provide valuable evidence for clinical decision-making.^1^ ^–^ ^4^ Most SRs are assembled from numerous scientific citations that are initially screened by human experts, a process that is both time-consuming and labour-intensive.^5^ ^–^ ^10^ Producing an SR typically takes an average of 67.3 weeks.^11^ The standard method for retrieving RCTs from databases often yields results where only 7% of the retrieved citations are real RCTs,^12^ making accurate identification of RCTs a crucial task when conducting SRs.^13^ Although machine learning (ML)^14^ ^,^ ^15^ models to accelerate the SR workflow by identifying RCTs have been developed, they are not widely used in practice due to concerns about their performance and practical benefits.^15^ ^,^ ^16^

Existing models^12^ ^,^ ^13^ ^,^ ^17^ ^–^ ^20^ have made considerable progress in reducing the workload and minimising missed RCTs, but their performance remains limited; precision is often sacrificed to ensure minimal missing, resulting in limited workload savings.^12^ ^,^ ^13^ ^,^ ^17^ For example, Thomas et al.^13^ reported high recall (0.99) but low precision (0.08) using a Support Vector Machine (SVM) on the Clinical Hedges dataset. Similarly, Marshall et al.^12^ obtained comparable results with a convolutional neural network model. More recently, BERT-based models like BioBERT have improved performance, achieving an F1 score of 0.91, which highlights the effectiveness of deep learning in RCT identification.^20^ Hybrid approaches that combine crowdsourcing with ML have also been explored to enhance efficiency.^17^ Ensemble methods, which combine SVM with PubMed publication-type prediction scores, have achieved a recall of 0.99 and precision of 0.21, demonstrating advances in RCT identification through ML and integrated techniques.^12^

A stacked ensemble approach, which feeds the outputs of several different fine-tuned BERT models into a Light Gradient Boosting Machine (LightGBM)^21^ classifier may enhance RCT identification, achieving improved precision and a recall rate as high as 0.99. The transformer architecture^22^ of BERT, which underpins models like GPT and ChatGPT, supports advanced text understanding capabilities. Its pre-training^23^ on diverse corpora allows for effective fine-tuning and robust cross-language capabilities, which are essential for text classification tasks required in RCT identification.^20^ The stacked ensemble method^24^ is particularly beneficial as it can detect complex patterns in the data that a single model might overlook, thereby improving overall predictive performance. The combination of LightGBM with BERT’s advanced text representation capabilities proves highly effective in text classification tasks, including the detection of fake news.^25^

Furthermore, the practical benefits of ML in identifying RCTs for title and abstract screening in SRs are often questioned by practitioners due to concerns^26^ about the ‘black-box’ nature of ML. This opacity can obscure the decision-making process, impacting transparency, and reliability, and potentially risking the exclusion of key evidence. SRs typically require at least two reviewers during abstract screening to ensure comprehensiveness^27^ ^–^ ^29^; however, this process is time-consuming and labour-intensive. ML-assisted screening might address these challenges, potentially replacing the need for a human reviewer.

Few studies have explored the impact of tool-assisted manual screening on practice.^26^ ^,^ ^30^ ^–^ ^32^ Clark et al.^30^ reported that an SR could be completed within 2 weeks using a suite of tools, such as the Word Frequency Analyzer and the Polyglot Search Translator for searching, as well as the SRA Helper and RobotSearch for screening. Their subsequent study^31^ demonstrated that teams using these tools saved 70% of the time on six specific tasks compared to those working manually. Perlman-Arrow et al.^32^ conducted a real-world study on the impact of ML assistance, reporting a 45.9% reduction in the time spent on title and abstract screening compared to manual methods. These studies highlight the advantages of tool-assisted SRs; however, the presence of various non-ML tools makes it challenging to discern the specific practical impact of ML on the effectiveness of SRs.

This study aims to (1) develop an ensemble learning model to identify RCTs, (2) evaluate its practical impact, and (3) provide recommendations for ML-assisted manual screening to facilitate the rapid conduct of SRs.

## 2 Methods

**Figure 1:**
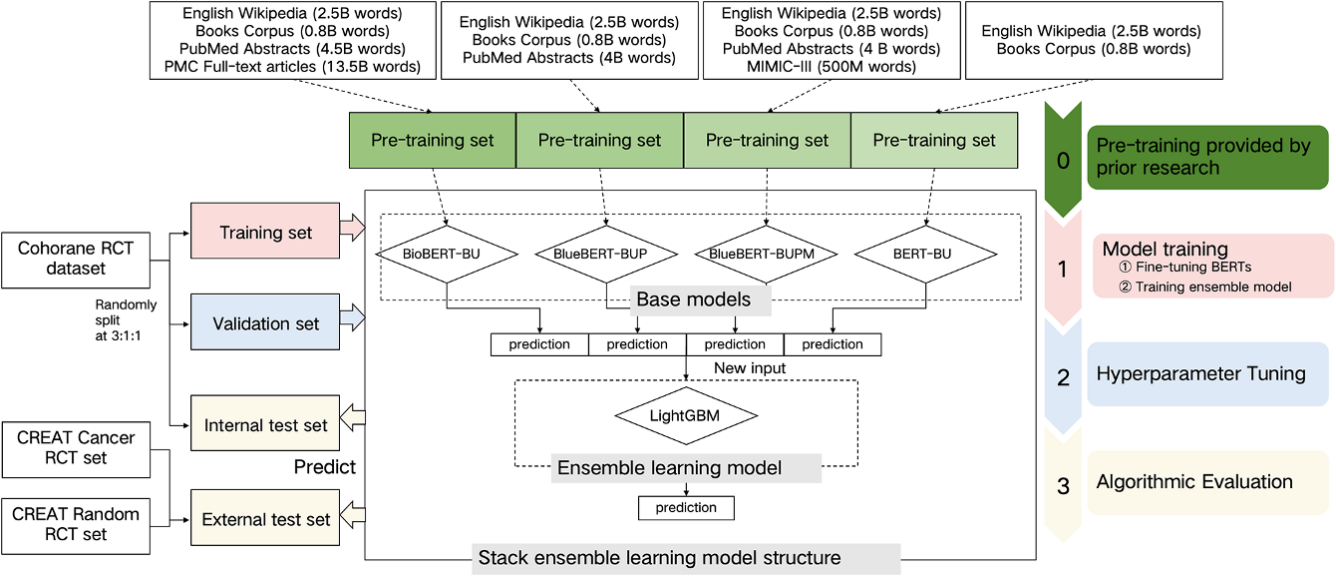
Model Frame.

We developed and internally evaluated an ensemble learning model using the Cochrane Crowd RCT dataset. The model was then externally evaluated on two RCT datasets, which included annotations with screening time. Following this, we assessed the impact of our model in two practical ML-assisted manual screening scenarios using these newly annotated datasets. The model framework is illustrated in Figure 1.

### 2.1 Data sources

#### 2.1.1 Development and internal evaluation dataset

We randomly divided the Cochrane Crowd RCT dataset into three subsets—training, validation, and internal test sets—at a ratio of 3:1:1 for model training, hyperparameter tuning, and internal performance evaluation, respectively. The dataset comprised 83,968 citations, including IDs, titles, and abstracts, with 7,356 RCTs (8.8%) and 76,612 not-RCTs (91.2%). The Cochrane Crowd RCT dataset is a robust and diverse resource, sourced from a global community of volunteers who contribute to identifying and classifying RCTs from bibliographic sources such as Embase, PubMed, and ClinicalTrials.gov. The crowd-based annotation process involves volunteers reviewing research study descriptions, with decisions made by at least two reviewers to determine whether the citations describe RCTs or not.^33^

#### 2.1.2 Datasets for external evaluation and assessment of practical impact

To illustrate the practical impact of ML compared with manual screening, we annotated new datasets, as no existing RCT datasets provided screening times and results for each reviewer. We annotated two independent RCT datasets, ensuring no overlap between them, with each citation including screening time and results (see the section “External and practical evaluation datasets” of the Supplementary Material for further details). These external test datasets were distinct from the Cochrane RCT dataset, ensuring an unbiased evaluation of the model’s performance on data not included in the original training or validation process. Each dataset comprised 5,000 citations (published between 2013 and 2022 in PubMed) in the form of titles and abstracts. One dataset, randomly selected without specific qualifying topics, was named the CREAT random RCT dataset, while the other, randomly selected from abstracts containing cancer-related topics, was named the CREAT cancer RCT dataset. CREAT represents our research team CREAT (Clinical Research, Evaluation and Translation). Standard manual screening was conducted on these datasets as the gold standard. Paired reviewers independently screened each citation in parallel for titles and abstracts, with a third trained reviewer resolving any disagreements.

### 2.2 Model development

We developed an ensemble learning model that integrates multiple base models to identify RCTs in SRs (see the section “Model development” of the Supplementary Material for further details). The ensemble model employed was LightGBM. The base models consisted of four BERT variants, all with the same structure and uncased vocabulary list but pretrained on different datasets, resulting in varying initial parameters. These models included BioBERT-BU,^34^ BlueBERT-BUP,^35^ BlueBERT-BUPM,^35^ and BERT-BU^23^ (see the section “Detail of different pretrained datasets of BERT models” in the Supplementary Material). The model development proceeded in three stages. The first stage involved fine-tuning the four BERT models using citation text as features to identify RCTs on the same training set. The second stage entailed training LightGBM with the classification results from the four BERT models as features on the training set. In the final stage, we fine-tuned the hyperparameters of LightGBM, including learning rate, tree complexity, and regularisation, to optimise the area under the curve (AUC) during cross-validation on the training set. After optimisation, we calibrated the cutoff on the validation set, prioritising a high recall (0.99) while maximising precision based on the precision-recall curve, to ensure comprehensive identification of all true RCTs while minimising workload.

### 2.3 Model evaluation

#### 2.3.1 Algorithmic evaluation

*Algorithmic evaluation: prior to cutoff adjustment*. We performed an algorithmic evaluation of our model (balance) with a 0.5 cutoff to assess its capability to enhance RCT identification. Before adjusting the cutoff, we evaluated the base BERT models and introduced an ensemble voting method *Vote* on the internal test set for comparison. To identify RCTs, the voting method *Vote* generates a score by averaging the prediction probabilities. For this evaluation, the voting method *Vote* (balance) used a 0.5 cutoff. We used standard manual screening as a benchmark to evaluate our model, applying both ML evaluation metrics (precision, recall, and F1 score) and epidemiological metrics (sensitivity and specificity). Recall and sensitivity are included to reflect the dual terminology of ML and epidemiology, despite being equivalent measures. Further details of these metrics can be found in the section “Algorithmic evaluation” of the Supplementary Material.

*Algorithmic evaluation: post cutoff adjustment*. After hyperparameter tuning, we identified the optimal cutoff value for our model using the validation set and uniformly applied this value to evaluate model performance on both internal and external test sets. This evaluation aimed to demonstrate the model’s potential to improve the RCT screening process, focusing on enhancing precision while maintaining a high recall rate of 0.99, which is crucial for capturing the maximum number of relevant RCTs.

To validate the model’s robustness across diverse data environments, we assessed its performance on both internal and external test datasets. With the adjusted cutoff value, we benchmarked our model against two established methods: the voting method *Vote* and a high-recall SVM^13^ model (details of which are provided in the section on “Model evaluation” of the Supplementary Material). The voting method *Vote* modified the cutoff value on the validation set, prioritising a high recall (0.99) while maximising precision based on the precision-recall curve, to ensure comprehensive identification of all true RCTs while minimising workload. These comparisons provided performance baselines, using both algorithmic and practical evaluation metrics (e.g., workload saving; further details of “Practical evaluation” are shown in the Supplementary Material).

#### 2.3.2 Evaluation of practical impact

**Figure 2:**
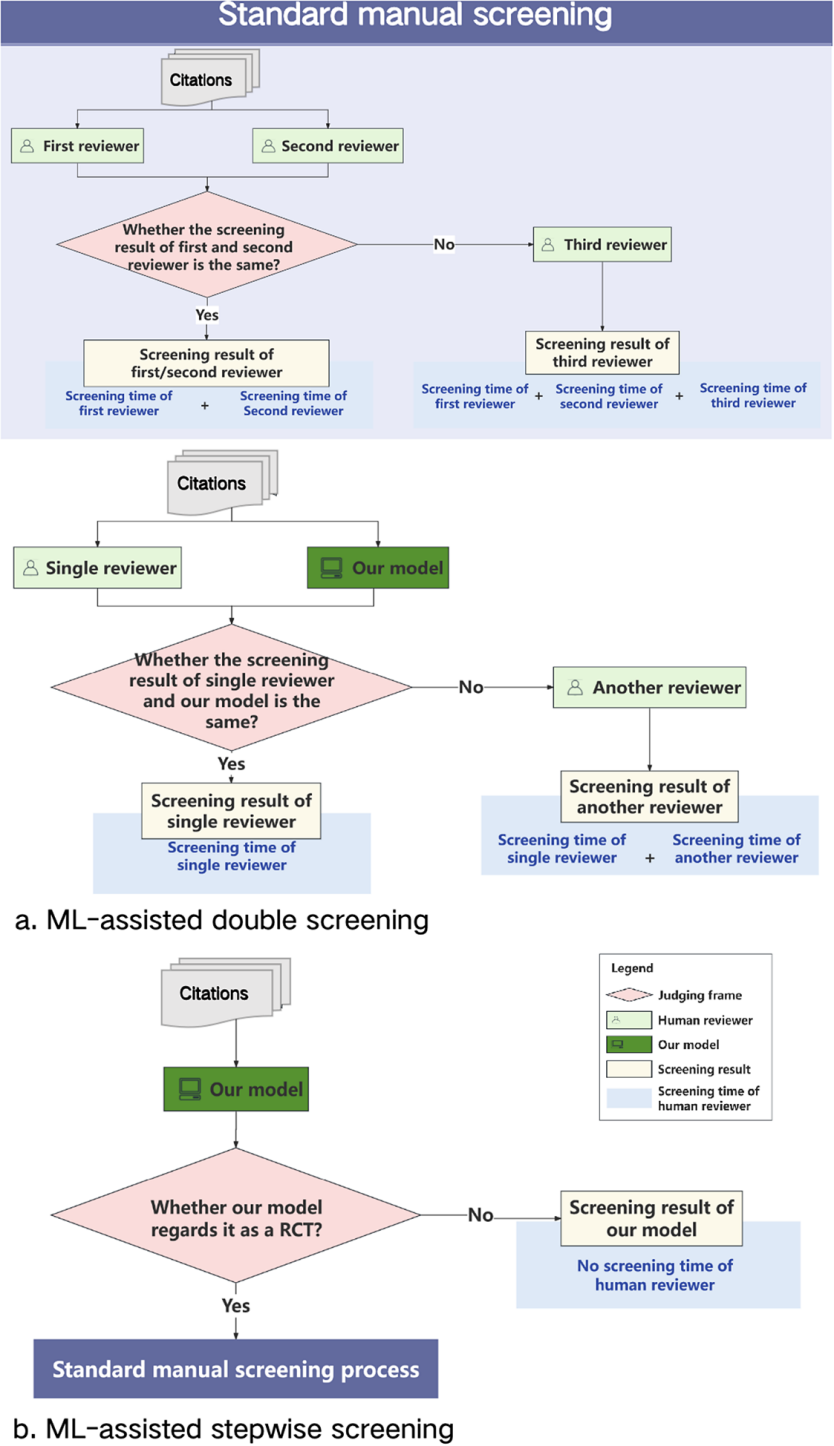
Overview of workflow and labour time in different screening scenario.

We assessed the practical impact of our model on the external test sets across two potential ML-assisted manual screening scenarios: ML-assisted double screening (Figure 2a) and ML-assisted stepwise screening (Figure 2b). Using standard manual screening as a benchmark (Figure 2), we established reference points for evaluating practical impact in terms of accuracy (with a focus on epidemiological metrics, particularly sensitivity) and labour saving (including labour time per scenario and labour time saving, detailed in the section “Labour saving” of the Supplementary Material).

*Standard manual screening scenario as the benchmark*. In the standard manual screening scenario, the process involves paired reviewers independently screening citations in parallel. When their results are consistent, the RCT identification result is taken from the first reviewer. If their results are inconsistent, a third reviewer resolves the disagreement.

We used standard manual screening as a benchmark to calculate both algorithmic and practical evaluation metrics across two separate external test sets. As illustrated in Figure 2, labour time is measured as the cumulative time taken by the paired reviewers. If the paired reviewers’ results are inconsistent, the labour time is increased by the duration required for a third reviewer to mediate and resolve the disagreement.

*ML-assisted double screening scenario*. In the ML-assisted double-screening scenario, the process is as follows: the ML model and a single reviewer independently screen citations in parallel. When their results are consistent, the RCT identification result is taken from the single reviewer. When their results are inconsistent, a third reviewer resolves the disagreement. As illustrated in Figure 2, the labour time is measured as the cumulative time taken by the single reviewer. If the single reviewer’s results do not align with the ML model’s, the labour time is increased by the duration required for a third reviewer to mediate and resolve the disagreement.

To enhance the reliability of the efficacy evaluation for the ML-assisted double screening method, we conducted two independent tests on the same external test sets, each with a different single reviewer working in parallel with the ML model. Any disagreements were resolved by a third reviewer. We simulated the performance of the single reviewer by using outcomes from one of the reviewers involved in the standard screening process.

Additionally, we performed two tests of the single reviewer screening scenario on the same sets to compare the effects of ML-assisted double screening. For each test, we evaluated the performance of the single reviewer using outcomes from the single reviewer involved in the ML-assisted double screening. The labour time for the single-reviewer screening scenario was determined by aggregating the time spent by that reviewer.

*ML-assisted stepwise screening scenario*. In the ML-assisted stepwise screening scenario, the process involves two steps. In the first step, the ML model screens all citations and flags potential RCTs. In the second step, human reviewers conduct standard manual screening of the flagged citations.

We applied this screening method once to two separate external test sets. The performance of standard manual screening on the flagged citations was assessed using outcomes from the standard screening process. As depicted in Figure 2, the new labour time reflects the time spent on manual screening of citations identified as RCTs by the ML model.

## 3 Results

### 3.1 Algorithmic evaluation

#### 3.1.1 Algorithmic evaluation: prior to cutoff adjustment

At this stage, prior to any adjustment of the cutoff, our model demonstrated strong algorithmic performance. It achieved a recall of 0.902, a precision of 0.953, and an F1-score of 0.927 on the internal test dataset from the Cochrane Crowd RCT dataset. Our model outperformed all baseline models (see Table 1), with an F1-score of 0.927 compared to 0.922 for the best baseline models, and a recall of 0.902 compared to 0.889 for the best baseline models.

**Table 1:** Algorithmic evaluation of models on the internal test from Cochrane Crowd RCT dataset

	Total		Algorithmic evaluation			
Model	citations	RCTs	Specificity	Recall/sensitivity	Precision	F1 score
BioBERT-BU	16,795	1472	**0.996**	0.875	0.953	0.912
BlueBERT-BUP			0.995	0.874	0.946	0.909
BlueBERT-BUPM			0.993	0.889	0.929	0.909
BERT-BU			0.994	0.889	0.938	0.912
*Vote* (balance)			**0.996**	0.889	**0.958**	0.922
Our model (balance)			**0.996**	**0.902**	0.953	**0.927**

*Note*: The “total citations” column represents the total number of citations in the internal test dataset from Cochrane Crowd RCT dataset. The “RCTs” column indicates the number of citations that were identified as RCT in the internal test dataset from Cochrane Crowd RCT dataset. Bolded values are used to highlight key performance metrics that are particularly significant in the context of our study.

#### 3.1.2 Algorithmic evaluation: post cutoff adjustment

As shown in Table 2, after adjusting the cutoff, our model maintained a high recall rate of 0.99 while achieving precision scores of 0.353, 0.483, and 0.400 across the Cochrane, CREAT random, and CREAT cancer datasets, respectively. These precision scores significantly surpass those of the SVM and *Vote* methods, effectively reducing false positives. Additionally, our model delivered substantial workload savings, reducing the manual review effort by 75.3%, 73.9%, and 86.4% for the respective datasets. This demonstrates the model’s ability to streamline the RCT screening process by enhancing precision and efficiency while preserving a high recall rate of 0.99.

**Table 2:** Single Model Performance in RCT Identifying: Algorithmic evaluation and practical evaluation on the internal and external test datasets

				Algorithmic evaluation				Practical evaluation
Dataset	Model	Total citations	RCTs	Specificity	Recall/sensitivity	Precision	F1score	Workload saving (%)
Cochrane data	SVM	16,795	1472	0.567	0.994	0.181	0.306	51.8
	*Vote*			0.772	**0.995**	0.295	0.455	70.4
	Our model			**0.825**	**0.995**	**0.353**	**0.521**	**75.3**
CREAT random RCT data	SVM	5,000	632	0.520	**0.998**	0.231	0.376	45.5
	*Vote*			0.675	**0.998**	0.308	0.471	59.0
	Our model			**0.846**	0.997	**0.483**	**0.651**	**73.9**
CREAT cancer RCT data	SVM	5,000	338	0.646	0.976	0.167	0.285	60.4
	Vote			0.717	**0.997**	0.203	0.338	66.8
	Our model			**0.926**	0.994	**0.400**	**0.570**	**86.4**

*Note*: In Table 2, the bolded values highlight the improvements in sensitivity, specificity, and workload saving achieved by our model over existing methods like the SVM and Vote ensemble.

### 3.2 Evaluation of practical impact

**Figure 3:**
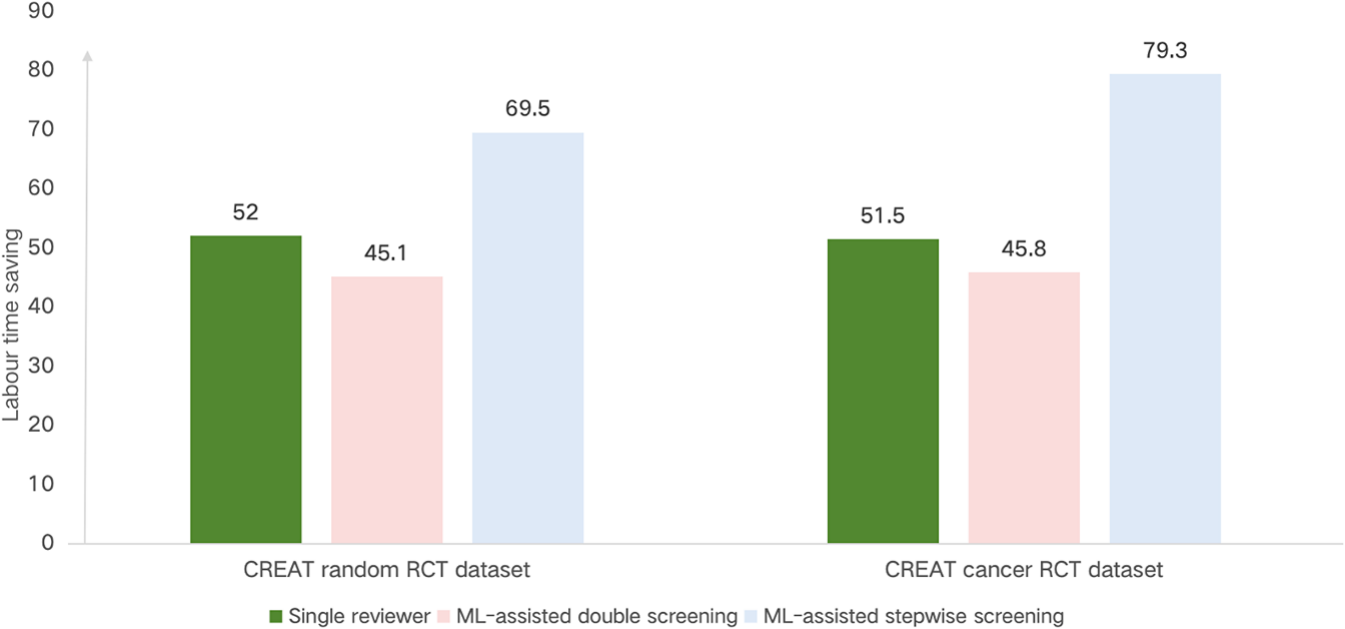
Comparative analysis of labor time saved (%) compared to standard manual screening across different screening scenarios.

The evaluation of the impact of ML in two practical scenarios is presented in Table 3. Figure 3 visually represents the labour time across different screening scenarios for the CREAT random and CREAT cancer RCT datasets.

**Table 3:** Comparative analysis of epidemiological metrics and labour time in screening scenarios for practical evaluation

		Epidemiological metrics		Labour saving	
		Average (first, second)		Average (first, second)	
Model	Dataset	Sensitivity	Specificity	Labour time per scenario(h)	Labour time saving (%)
Single reviewer	CREAT random RCT dataset	0.927 (0.973,0.881)	0.985 (0.980,0.990)	17.62 (17.37,17.87)	52.0 (52.7,51.3)
	CREAT cancer RCT dataset	0.911 (0.944,0.879)	0.988 (0.986,0.991)	17.77 (17.79,17.74)	51.5 (51.4,51.6)
	Average of these two datasets ^#^	0.919	0.987	17.70	51.7
ML-assisted double screening	CREAT random RCT dataset	0.998 (0.997,1.000)	0.986 (0.981,0.991)	20.17 (19.79,20.55)	45.1 (46.1,44.0)
	CREAT cancer RCT dataset	0.998 (0.997,1.000)	0.990 (0.987,0.992)	19.84 (19.74,19.94)	45.8 (46.1,45.6)
	Average of these two datasets ^#^	0.998	0.988	20.00	45.4
ML-assisted stepwise screening	CREAT random RCT dataset*	0.997	1.000	11.20	69.5
	CREAT cancer RCT dataset*	0.994	1.000	7.59	79.3
	Average of these two datasets ^#^	0.996	1.000	9.39	74.4

*Note*: Average (first, second): Since we conducted two tests of single reviewer screening and ML-assisted double screening, this represents the result from the first test, the result from the second test, and average values of the results from these two tests. ‘*’: Because we only conducted one test of single ML-assisted stepwise screening, there is only one set of results. The symbol ‘#’ represents the mean performance across two datasets.

#### 3.2.1 Standard manual screening scenario as the benchmark

In the standard manual screening scenario, the results indicated that screening 5,000 citations, which included 632 RCTs in the CREAT random RCT dataset and 338 RCTs in the CREAT cancer RCT dataset, took 36.71 h for the random dataset and 36.63 h for the cancer dataset, respectively. Individually, a reviewer took an average of 12.71 s to screen a citation in the random dataset and 12.81 s in the cancer dataset.

#### 3.2.2 ML-assisted double screening scenario

We conducted two tests of ML-assisted double screening on the CREAT cancer RCT dataset and the CREAT random RCT dataset. Our model achieved a higher recall rate than single reviewers, with an average improvement from 0.919 to 0.998. Specifically, the average recall was 0.998 for ML-assisted double screening versus 0.927 for single reviewer screening on the CREAT cancer dataset, and 0.999 versus 0.911 on the CREAT random RCT dataset. However, this improvement in recall resulted in a modest reduction in labour time savings, averaging 45.3% compared to 51.7% for the single reviewer scenario. In detail, the labour time savings were 45.1% versus 52.0% for the CREAT random dataset and 45.8% versus 51.5% for the CREAT cancer dataset. Figure 3 illustrates these findings, with the labour time savings for ML-assisted double screening denoted by a relatively shorter bar, indicating a more efficient labour time requirement compared to the standard screening method.

#### 3.2.3 ML-assisted stepwise screening scenario

In the ML-assisted stepwise screening scenario, our model performed comparably to standard manual screening while achieving substantial labour time savings, averaging 74.4%. Specifically, the savings were 69.5% for the CREAT random RCT dataset and 79.3% for the CREAT cancer dataset. Figure 3 highlights the labour time savings for this scenario, showing ML-assisted stepwise screening with the shortest bar, indicating it as the most time-efficient method among those evaluated.

## 4 Discussion

### 4.1 Recommendations for applying ML in identifying RCTs for rapid title and abstract screening in SRs

Based on the varying requirements of different application scenarios, we offer recommendations for 2 ML-assisted manual screening approaches, as outlined in Table 4.

**Table 4:** Recommendations for SR reviewers to apply ML-assisted manual screening to identify RCTs for rapid title and abstract screening in SRs

Application scenarios	Limited the number of reviewers	Limited time
Application	ML-assisted double screening	ML-assisted stepwise screening
Implementation	Replace one human reviewer with ML in standard manual screening	Step 1: Use the model to screen all the citations Step 2: Conduct standard manual screening on the citations included by ML
Effect	Reduces labour time by nearly half Higher recall than a single human reviewer	Reduces labour time by nearly three-fourths Performance close to that of standard manual screening
Limitation	When human reviewers and models misclassify simultaneously, real RCTs are lost.	When the model misclassifies RCTs, real RCTs is lost.
Suggestions	Human reviewers should have extensive screening experience.	Randomly sample the citations excluded by the model to check for missing.

When the number of reviewers available for SRs is limited, ML-assisted double screening is a promising solution. This method reduces labour time by half and outperforms a single reviewer. Reviewers engaged in double screening with ML should have extensive screening experience to minimise the risk of missing real RCTs. Our model is designed to enhance precision while maintaining a high recall of 0.99, thus ensuring minimal omission of real RCTs and reducing workload. Consequently, the individual consistency between our model and human reviewers in double screening is not very high, but it effectively serves as a useful alert mechanism.

When time constraints are a significant factor in conducting an SR (e.g., for rapid reviews^36^), ML-assisted stepwise screening can reduce labour time by three-fourths while performing comparably to standard manual screening. To prevent missing real RCTs, random sampling of citations excluded by the model should be conducted.

### 4.2 Main findings and implications

The main findings of this study are as follows. (1) The LightGBM model, which integrates multiple BERT models, demonstrated superior performance in identifying RCTs compared to the existing high-recall SVM model. It reduced the workload significantly while maintaining similar high recall in both internal and external tests. (2) The model showed promising results in 2 ML-assisted manual screening scenarios. In the ML-assisted double screening scenario, it reduced labour time by half and increased the recall compared to a single reviewer. In the ML-assisted stepwise screening scenario, it cut labour time by three-fourths while achieving performance comparable to standard manual screening. (3) Recommendations for ML-assisted manual screening in rapid title and abstract screening for SRs were provided, highlighting the potential benefits of integrating ML techniques in these processes.

#### 4.2.1 Improved workload savings with minimal missing cases in RCTs identification in SRs

Our results demonstrate that the ensemble model, which integrates various BERT models, consistently provides a higher recall than individual BERT models. The different BERT base models, pre-trained on diverse datasets, capture distinct aspects of the underlying patterns and discriminative properties. This allows ensemble learning to extract and interpret semantics from citations more effectively, resulting in more accurate RCT identification.

Previous studies^13^ ^,^ ^18^ ^,^ ^3^ ^,^ ^7^ have employed SVMs to reduce workload and minimise missing cases, but these approaches often overlooked semantic information, limiting their effectiveness. By adjusting the cutoff value of our model to achieve a sensitivity/recall of 0.99, we improved performance significantly compared to existing high-recall SVM models. Our modified model achieved a three-fourths reduction in workload while maintaining the same level of sensitivity (0.99) in both internal and external evaluations. The BERT ensemble leverages advanced language understanding capabilities to capture the semantic richness and contextual nuances of medical literature. Its deep learning framework facilitates sophisticated feature extraction and representation, which is crucial for complex classification tasks such as RCT identification. Additionally, the ensemble’s combined learning approach addresses individual model weaknesses, enhancing overall accuracy.

#### 4.2.2 Impact of our model in two practical scenarios

Our model demonstrated a promising impact on newly annotated RCT datasets in two potential ML-assisted manual screening scenarios: ML-assisted double screening and ML-assisted stepwise screening.

*ML-assisted double-screening scenario*. In this scenario, our model achieved a higher recall than a single human reviewer while reducing labour time by nearly half. Although single-reviewer screening could accelerate the process, it is not recommended by guidelines due to the increased risk of missing studies and potential bias.^28^ ^,^ ^29^ ML-assisted double screening offers a solution by improving recall while maintaining similar labour time. Discrepancies between the single reviewer’s results and those of the ML model are resolved by a third-party reviewer, ensuring no RCTs are overlooked.

For cases missed by ML-assisted double screening, a thorough review of full texts confirmed that none were RCTs. These omissions were due to the information available in titles and abstracts being insufficient to accurately determine the study type, thus highlighting the limitations of relying solely on these sources for classification.

*ML-assisted stepwise screening scenario*. The ML-assisted stepwise screening scenario performed comparably to standard manual screening, with a labour time saving of three-fourths. This approach significantly reduces the workload by excluding citations through ML screening before manual review. However, there is a risk of missing important information, making it crucial to use a model with high recall to ensure that real RCTs are not overlooked. This scenario is particularly beneficial for rapid reviews when time is constrained.

### 4.3 Strengths

A key strength of our study is that the developed model outperformed the high-recall SVM model across diverse datasets, achieving greater workload reduction with minimal missing cases. We also annotated two new RCT datasets, demonstrating the practical benefits of ML-assisted manual screening. To our knowledge, no previous studies have directly compared the impact of ML on labour time and recall improvement with human reviewers. Our findings provide reviewers with insights into the actual benefits of ML. Additionally, we offer recommendations to assist reviewers in applying ML effectively, ensuring the timely completion of SRs.

### 4.4 Limitations

This study has several limitations. Firstly, our model is limited to identifying English RCTs and may not apply to languages with non-Latin alphabets due to the constraints of BERT’s tokenizer. Additionally, the model’s complexity requires substantial computational resources, resulting in longer training and prediction times compared to a single BERT model or an SVM model. However, a notable advantage is the reduced need for data pre-processing compared to SVM models, which enhances efficiency and reduces computational demands. There is also a risk of performance overestimation due to overlap with BERT’s pre-training datasets, particularly with external PubMed datasets from 2012–2023. Finally, it is crucial to validate the applicability of ML-assisted strategies and their recall thresholds with new data to ensure their continued effectiveness, especially when applied to datasets that differ significantly from the Cochrane RCT dataset used in our study.

## 5 Conclusions

In conclusion, we developed an ensemble learning model that integrates multiple BERT models. This model effectively identifies RCTs for the rapid title and abstract screening in SRs, significantly reducing workload and minimising missed cases. Our findings suggest that ML-assisted double screening is a promising solution when the number of reviewers is limited. Additionally, ML-assisted stepwise screening proves valuable when time constraints are present in producing SRs.

## Supporting information

Qin et al. supplementary material Qin et al. supplementary material

## Data Availability

The annotated data and source code are available on GitHub (https://github.com/RuringQinXuan/RCT_recognition_lightgbm.git).

## Supplementary material

To view supplementary material for this article, please visit http://doi.org/10.1017/rsm.2025.3.
