# Supplementary material for: Machine learning for identifying randomised controlled trials when conducting systematic reviews: Development and evaluation of its impact on practice
Source: Res Synth Methods. 2025 Mar 21;16(2):350–63. doi: 10.1017/rsm.2025.3 (PMC12527483; doi:10.1017/rsm.2025.3)
Supplement: Qin et al. supplementary material [file S1759287925000031sup001.docx]

## External and practical evaluation datasets

### Detail of annotation

In three steps, we annotated two RCT datasets as external and practical evaluation datasets, namely the CREAT random RCT dataset and the CREAT cancer RCT dataset. First, we retrieved RCT title-and-abstract citations of created between January 1, 2012, and January 1, 2023, in the PubMed database according to the following search strategy:

((((randomized controlled trial[pt] OR controlled clinical trial[pt] OR randomized[tiab] OR placebo[tiab] OR drug therapy[sh] OR randomly[tiab] OR trial[tiab] OR groups[tiab]) NOT (animals[mh] NOT humans[mh])) NOT (Review[Create Type])) NOT (Books[Create Type] AND Documents[Create Type])) AND (("2012/01/01 "[Date - Create] : " 2023/01/01 "[Date - Create])).

Here's what search components represent in the given search term:

[pt]: This stands for "Publication Type" and is used to filter articles by their type, such as "randomized controlled trial" or "controlled clinical trial".

[tiab]: This indicates "Title/Abstract" and is used to search for terms that appear in both the title and abstract of the articles.

[sh]: This represents "Subheading" and is used for searching terms that are categorized under specific subheadings in the MeSH database.

[mh]: Stands for "MeSH Terms" and is used to search for terms that are MeSH descriptors, which are controlled and standardized keywords assigned to articles in PubMed.

[Create Type]: This is a PubMed filter that specifies the type of document, such as "Review", "Books", or "Documents". It helps in excluding certain document types from the search results.

[Date - Create]: This is used to filter articles based on their creation date in the PubMed database, allowing the search to be limited to a specific time frame.

Then, we randomly selected 5000 citations as the CREAT random RCT dataset and randomly selected 5000 citations with ‘cancer’ in the title or abstract as the CREAT cancer RCT dataset. Finally, we organized a team of seven trained epidemiology students to conduct the standard manual screening to identify RCTs on these two datasets, where paired reviewers independently screened each citation for titles and abstracts, with a third reviewer resolving any disagreements.

## Model development

### Detail of model development

The model is an ensemble learning model integrating four different base models.

**Four base models**

We began with BERT models initialized from BioBERT-BU, BlueBERT-BUP, BlueBERT-BUPM, and BERT-BU, provided by prior researches. Subsequently, we fine-tuned each of these BERT models for the task of identifying RCTs.

**Ensemble learning model**

We used a stacking ensemble method to harness the strengths of multiple fine-tuned BERT models. The LightGBM functioned as an RCT classifier, utilizing the outputs from the BERT models as new inputs. By applying gradient boosting in LightGBM, we efficiently combined new inputs, refined the features, and optimized the classification process to accurately distinguish between RCTs and not-RCTs. The ensemble's output was a two-dimensional vector representing the possibility of a positive label (RCT) and a negative label (not-RCT).

Detail of different pretrained datasets of BERT models

The BERT-BU was pre-trained on English Wikipedia (2.5B words) and BooksCorpus (0.8B words). BioBERT-BU was pre-trained on a diverse corpus including English Wikipedia (2.5B words), BooksCorpus (0.8B words), PubMed Abstracts (4.5B words), and PMC Full-text articles (13.5B words) totaling over 2.47 million steps. BlueBERT-BUP is a variant of BERT-BU that was further pre-trained on the PubMed Abstracts (4B words) dataset for an additional 500 million training steps. BlueBERT-BUPM is another variant of BERT-BU that was further pre-trained on the PubMed Abstracts (4B words) for 500 million training steps and MIMIC-III (500M words) for 0.2 million training steps.

## Model evaluation

**The baseline SVM model**

The SVM model employed a bag of words text representation, utilizing unigrams and bigrams, and applied TF-IDF weighting to enhance performance. Data preprocessing involved the elimination of common stopwords and the adoption of a bag-of-words model to represent the text data effectively. The SVM implemented an oversampling strategy to address class imbalance and trained on a 280,620 title-abstract records from Embase, meticulously labeled by the Cochrane Crowd. The output was calibrated using logistic regression for interpretable probability scores, and a threshold was set to ensure 99% recall, crucial for minimizing missed real RCT studies in SRs.

### Definition of evaluation metrics

**Algorithmic evaluation**

**ML evaluation metrics**

Precision is the ratio of the correctly predicted RCTs to the citations the model predicted as RCTs. Recall (sensitivity) is the ratio of correctly predicted RCTs to the total number of RCTs. F1 score is the harmonic mean of precision and recall that provides a balanced evaluation of a classification model's performance.

**Epidemiology metrics**

Sensitivity and recall are included to reflect the dual terminology of machine learning and epidemiology, despite being equivalent measures. Specificity is the ratio of the number of not-RCTs correctly predicted as not-RCTs to the total number of not-RCTs.

**Practical evaluation**

Workload saving is as defined as the ratio of the number of citations predicted as not-RCTs to the total number of citations.

**Labour saving**

The labour time per Scenario was the sum of the time spent by all human reviewers on screening the dataset under the Scenario. The labour time saving was calculated as one minus the ratio of the new labour time to the labour time of the standard manual screening, between 0 and 1. A higher labour time saving indicates a better practical performance of the ML.
